# Hotels’ Eco-Friendly Physical Environment as Nature-Based Solutions for Decreasing Burnout and Increasing Job Satisfaction and Performance

**DOI:** 10.3390/ijerph17176357

**Published:** 2020-09-01

**Authors:** Jongsik Yu, Antonio Ariza-Montes, Felipe Hernández-Perlines, Alejandro Vega-Muñoz, Heesup Han

**Affiliations:** 1 College of Business, Cheongju University, 298 Daeseong-ro, Cheongwon-gu, Cheongju-si 28503, Korea; andyjs.yu@gmail.com; 2 Department of Management, Universidad Loyola Andalucía, 14004 Cordóba, Spain; ariza@uloyola.es; 3 Department of Business Administration, University of Castilla-La Mancha, 45071 Toledo, Spain; Felipe.HPerlines@uclm.es; 4 Faculty of Business Administration, Universidad Autónoma de Chile, Santiago 7500912, Chile; alejandro.vega@uautonoma.cl; 5 College of Hospitality and Tourism Management, Sejong University, 98 Gunja-Dong, Gwanjin-Gu, Seoul 143-747, Korea

**Keywords:** nature-based solutions (NBSs), green hotels, existing natural environments, burnout, job satisfaction, job performance

## Abstract

This study investigates the effect of the hotel’s nature-friendly environment on burnout, job satisfaction and job performance of hotel employees. A total of 11 hypotheses were set up to achieve the purpose of this study, and an empirical analysis was conducted based on 309 surveys collected from hotel employees. A total of 11 hypotheses were set to achieve the research goals, and an empirical analysis was conducted based on a total of 309 pieces of data collected from 320 hotel employees who are currently working in 11 hotels in South Korea. As a result, eight hypotheses were accepted and three were rejected. Specifically, it was found that the hotel’s nature-friendly environment reduced burnout in employees, and indirectly had a significant effect on job satisfaction and job performance. Therefore, the relationship between the variables presented was clearly demonstrated through the research results, and the purpose of this study was satisfactorily explained. The results are expected to be of great help to hotel employees and researchers in developing strategies to efficiently manage hotel employees through nature-based solutions (NBSs). Based on the results, the proposed theoretical and practical implications are discussed in detail in the discussion section.

## 1. Introduction

In this study, we focused on nature-based solutions (NBSs), which constitute part of the green spaces of a hotel, to decrease the burnout phenomenon that can occur on the job, as well as to improve employee satisfaction and performance, with a focus on the mental health, welfare, comfortable working environment, and stress recovery of hotel employees. Nature-based solutions (NBSs) refer to actions that are inspired or aided by nature and are defined as efficient and adaptive methods that can solve various environmental issues, while at the same time provide economic/social/environmental benefits [1]. Therefore, the reason for putting focus on the NBSs in this research is as follows. First, green spaces have positive effects on people’s mental and physical health as well as their well-being, and they have a great effect on improving the healing ability that aids in recovery from psychological distress, such as stress and depression [2,3]. Second, NBSs can provide economic, social, and environmental benefits by enriching people’s lives through human welfare improvement, the provision of emotional well-being, the provision of new business opportunities, etc., as well as by solving various social issues [1]. For these reasons, it is very appropriate to search for methods of improving the emotional well-being and working environments of employees through NBSs in the hospitality industry and especially in hotels, in which the role of human resources is very important. However, almost no research has been conducted on the emotional response of employees to NBSs in the hospitality industry. In particular, descriptions of the very positive effect of the exposure to nature-friendly environments on the mental health of employees and the influencing relationship on the satisfaction and job performance of employees through this effect are very limited. In addition, the alleviation of mental stress and emotional depression is a global public issue related to human welfare [4,5]. Recently, achieving mental health and emotional well-being through green environments has become an important lifestyle concept, and these factors can aid in improving performance through hotel employee satisfaction and furthermore, can greatly aid in productivity improvements and long-term profits for hotel corporations.

The job performance of frontline employees in the hospitality industry is a core concept in corporate development and long-term success strategies. Frontline employees are responsible for the most essential parts of the service provision and complaint-handling processes. In addition, as their attitudes and actions determine customers’ perceptions of service quality, frontline employees are considered very important entities [6]. As such, because frontline employees form the relationship between customers and quality, their loyalty toward the organization and customers is a predictive factor for sales effects and revenue growth and may be an even more important factor than customer loyalty toward the company. Especially in today’s highly competitive hospitality industry, maintaining and improving frontline employees’ job competence so that they can focus on their roles and maximize performance is a requisite factor for corporations. However, most managers argue that 70% of frontline employees are not able to work at their maximum potential [7]. Thus, much prior research has been conducted regarding the job performance of frontline employees, and such research will continue to be conducted in the future. Given the importance of frontline employees, the importance of the related research cannot be overemphasized. Therefore, the development of new and effective strategies that can maximize the job performance of hotel employees is a very natural aim of this research.

The impact of environmentally friendly environments on peoples’ lives is increasing [8,9]. Therefore, this research presents the following research objectives. Considering the growing effects of natural environments on human life, this study addressed the following objectives: (1) to test nature-based solutions developed for hotel employees by dividing the green areas in hotels into specially-designed green places/spaces within hotels and existing natural environments; (2) to test whether it is possible to minimize risk factors, such as burnout, in relation to mental health and wellness among hotel employees through NBS initiatives; (3) to test whether job performance and satisfaction in hotel employees can be improved by positive effects of NBSs; (4) to develop a new theoretical framework on the effects of NBSs on hotel employees by assessing the difference in outcomes of NBS applications between male and female employees. In the remainder of this paper, after the introduction, we briefly discuss the concept of the following variables, and then, we introduce the research methodology, including the scales and sample characteristics of the variables. Finally, we aim to discuss the implications and limitations based on the statistical results as well as directions for future research.

## 2. Review of the Literature

### 2.1. Nature-Based Solutions

Modern society is faced with extensive issues, such as unsustainable urbanization, health problems, declines and losses of natural resources, the pollution of the ecosystem (e.g., water, air, and soil pollution), climate change, and increased threats of natural disaster. As society searches for various solutions to these problems, NBSs are beginning to receive attention as a way to achieve desirable results, such as decreases in disaster risk, improvements to human welfare, and green growth [10]. NBSs have the potential to contribute to green growth, achieve a future-oriented society, improve human welfare, provide new business opportunities, and solve various social problems sustainably [1]. Types of NBSs include ecosystem restoration, the greening of grey surfaces (e.g., green rooftops, greened brownfields, and green walls), integrated broad-scale climate change mitigation, and adaptation measures (e.g., afforestation, constructed wetlands, and natural flood control) [1,10]. Recent research on NBSs can largely be divided into three categories: (1) NBSs related to green infrastructure [11,12,13]; (2) NBSs for the alleviation of and adaptation to climate change [14]; (3) NBSs related to ecosystem services [11,15,16]. As shown, NBS research related to human health and welfare mostly takes a cursory approach, with most studies discussing the fundamental ideas related to the aforementioned three concepts [17]. This research shows that NBSs are mostly focused on environmental and ecological issues. However, the healthy lives of humans are affected by the surrounding social and physical environments, and especially when people and the natural environment form a complementary relationship, the environment can improve people’s mental and physical health. Therefore, NBSs play a very important role in people’s happiness, and there is a corresponding need for research on the benefits to be gained from NBSs and their effects.

### 2.2. Mental Health Benefits and Nature

Depression and stress, which are among the most common mental illnesses, are considered the greatest health issues people face based on illness factors [18]. As such, depression and mental illnesses cause negative results, such as personal pain, significant economic costs, social issues, and qualitative distortions in the quality of life and welfare. Furthermore, it has been shown that these outcomes increase the suicide rates of depression patients and are related to other mental illnesses [19]. A report from the World Health Organization (WHO) predicts that depression and mental stress will become the most burdensome diseases in the world after heart diseases by 2020 [20].

Recently, interest in the natural environment has continued to increase in search of methods to improve people’s mental health and recovery ability, and many studies are being conducted in the fields of economics, sociology, psychology, the environmental sciences [21]. In particular, many studies have been conducted in the last ten years on the effects of urban green environments and plant diversity on psychological welfare. The results of these studies show that as the natural environment includes more plentiful plants and water, mental health improves [22], and green environments in cities and rural areas both provide an essential resource for physical and mental health and have a positive effect on people’s well-being [5]. Natural environments have been proven to have a significant impact on recovery and recovery ability; more specifically, exposure to natural environments appears to have a very large impact on recovery related to mental symptoms, such as psychological distress, depression, and anxiety [2,3].

### 2.3. Stress Recovery Theory

People’s typical environments are being greatly impacted by rapid urbanization, industrial development, and damage to the natural environment. Negative changes to the environment have many negative effects, including continuous stress, and long-term exposure to an urban environment has a negative physical and mental impact [23,24]. More specifically, exposure to an urban environment through industrial development has been shown to be linked to obesity, high blood pressure, and diabetes [24], and the increase in stress due to traffic sound pollution deteriorates health and is related to depression [23]. According to a report by the WHO, the increase in stress is one of the main contributing factors to early death in modern society [25]. Thus, stress has a large impact on the health and welfare of most people.

The negative impact that stress has on people has led to research on various methods to relieve or recover from stress. Existing research emphasizes approaches to green environments, that is, the need for efforts to become closer to the natural environment [26]. The fact that natural environments can facilitate recovery from stress is well explained by stress recovery theory (SRT). According to SRT, natural environments are very important for people’s functional elements and provide great help in recovering from stress. Therefore, SRT can cure the negative impact of long-term exposure to urban environments through green landscaping, green interior design, green therapy, and so on [27]. These positive aspects of SRT are supported by the biophilia hypothesis. This hypothesis states that humans interact with nature and that they have an intrinsic tendency to become part of nature [28]. The interaction between humans and nature can be seen in the functional changes that occur regarding people’s physical and mental fatigue. According to Ulrich [29], nature improves people’s survival capacity by providing an opportunity to recover from mental or physical fatigue and other stress factors, and this recovery ability facilitates the solving of complex problems, which is an essential skill for the progressive development of humankind, and aids in maintaining and developing cognitive abilities, such as creativity. The fact that human cognitive ability and emotional recovery are greatly influenced by the natural environment has been proven by SRT. Therefore, the natural environment is essential for people to heal and recover from physical and mental fatigue, and that importance needs more emphasis.

### 2.4. Green Environments of Hotels

The physical environment refers to the environment where a service is delivered to customers and where interactions between customers and employees occur. This environment has an impact on the attitude of the employee that is serving the customer, and it is considered an artificial environment that the company can control [30]. It is widely known that physical environments are deeply related to the responses and behaviors of customers and employees [31]. In addition, many researchers agree that environmental stimuli and atmospherics induce responses from customers and employees (e.g., approach or avoidance) [30,32]. In recent studies, many researchers describe surrounding environments that include elements of the five senses, such as smell and sound, as intangible environmental stimuli that cause unconscious sensory perceptions, reactions, and behavior [32,33,34].

As nature-friendly environments improve the health and comfort of indoor residents, attention to indoor environmental quality is gradually increasing [35]. Indoor environmental quality refers to the qualitative standards of the indoor environment (e.g., temperature, air quality, smell, indoor interior design, decorations, specially designed green places, space layout, and the existing natural environment). Nature-friendly environments are more emphasized in the luxury segment, with special interest in nature-friendly environments in hotels, which can largely be divided into two categories: (1) specially designed places (e.g., lobbies, rooms, restaurants, toilets, and spas) and (2) the existing natural environment (e.g., mountains, rivers, seas, lakes, and parks surrounding the hotel). Nature-friendly environments have been shown to improve the economic profits of a company and improve work efficiency through measures such as improved employee productivity, reduced absenteeism, decreased health insurance costs, and decreased compensation costs [36,37]. In addition, nature-friendly environments in buildings like hotels tend to facilitate the circulation of air, improve air quality, make the air less dry, and reduce high temperatures [38].

### 2.5. Burnout

Burnout was first conceptualized by Freundenberger [39], a pathologist, based on the fact that employees in customer service jobs in the 1970s experienced physical and mental exhaustion due to unrealistic and excessive workloads. Since then, burnout has been perceived as a very serious and dangerous phenomenon, and it is known as the most dangerous phenomenon of the 21st century [40]. Past research has focused on identifying the causes of burnout, but many issues have been raised regarding these research results. However, this research was generalized with the development of the Maslach Burnout Inventory, developed by Maslach and Jackson [41]. Letier and Meecham [42] categorized burnout as emotional exhaustion, reduced personal accomplishment, and depersonalization. Emotional exhaustion refers to the drying up of emotion due to emotional contact with others, reduced personal accomplishment refers to the lack of feelings regarding the successful achievement of goals in relation to work with others, and depersonalization refers to non-emotional and cold responses toward people who need service and care, that is, the disadvantaged classes [42]. This classification is the most commonly used conceptual definition and measurement tool of burnout to date.

The burnout phenomenon manifests in mental and physical problems, such as serious psychological problems, prolonged stress, and excessive exhaustion, perceived by an organization’s members [43]. Although such phenomena can occur in anyone, it has been shown that employees who work in the service industry (e.g., hotels, restaurants, airlines, hospitals, etc.) experience high levels of burnout [44]. A representative group of people who are exposed to burnout in the service industry are hotel employees. Hotel employees experience emotions such as frustration and despair through frequent contact with customers in work situations, and they show such symptoms as mental and physical fatigue when processing service complaints [45]. For example, hotel employees’ service competency for customer satisfaction tends to fall due to night shift work, inconsistent work, non-specific days off, service failures that occur during service encounters, the psychological stress and mental pressure that occur during the complaint-processing process, and so on.

### 2.6. Job Satisfaction and Job Performance

Job satisfaction is defined as an enjoyable and positive emotional state regarding the organization and the job perceived by a member of an organization. It is an emotional response to the job, and it manifests through a comparison between actual and expected results [46]. As the experience and satisfaction gained through a job are transferred to satisfaction in an individual’s personal life, it has a large impact on the growth of a corporation based on an individual’s perceived life satisfaction and improved job performance [47]. In addition, job satisfaction is determined by the personal emotions felt by an individual while they perform their job [48]. An organization member’s positive emotional state increases individual productivity, maintains good physical and psychological health, and improves morale. These positive results of job satisfaction can create advantageous attitudes toward the organization (e.g., reduced turnover intent, increased self-efficacy, voluntary efforts, and the development of relationships between organization members) and can increase the performance of the company. Therefore, the job satisfaction of organization members is very important for ultimately achieving the financial and non-financial goals of a company.

Sonnentag and Frese [49] argue that a corporation requires employees with high job competency levels to achieve the organization’s goals and to be differentiated from, and be more competitive than, similar corporations. An examination of prior research shows that studies emphasize the direct role of employees in job performance and endeavor to find methods to maximize job performance [6,50]. More specifically, to improve job competency, the confidence and job satisfaction of organization members need to be improved, and from a psychological perspective, employee education (e.g., education programs that can aid in the rapid recovery from work stress and mental fatigue, work competence improvement programs to increase voluntary motivation, etc.) and work environment improvements (e.g., human or material resources required by the employee, the securing of green space for mental healing, etc.) are required [51]. When considering the importance of job performance, implementing these two methods in a complementary fashion can greatly aid in improving job performance [52].

### 2.7. Impact of Green Enviroments of Hotels on Burnout

Green natural environments provide satisfaction and value due to the emotional attachment that forms between people and the natural environment [53]. That is, when people come in contact with the natural environment, they feel emotional happiness, and they perceive an escape from various social activities [21]. Green environments have been shown to have clear advantages in providing the basic elements of mental health, including the recovery process from stress or mental illness [18]. Considering these results, we can say that nature-friendly environments (e.g., specially designed places and existing natural environments) have a significant effect on the prevention of and recovery from emotional stress. Therefore, in this study, we set the following hypotheses based on prior research.

**Hypothesis 1** **(H1).** *Specially designed green places and spaces (SDGPs) within hotels will decrease emotional exhaustion (EE).*

**Hypothesis 2** **(H2).** *Specially designed green places and spaces (SDGPs) within hotels will decrease reduced personal accomplishment (RPA).*

**Hypothesis 3** **(H3).** *Specially designed green places and spaces (SDGPs) within hotels will decrease depersonalization (DEP).*

**Hypothesis 4** **(H4).** *Existing natural environments (ENE) will decrease emotional exhaustion (EE).*

**Hypothesis 5** **(H5).** *Existing natural environments (ENE) will decrease reduced personal accomplishment (RPA).*

**Hypothesis 6** **(H6).** *Existing natural environments (ENE) will decrease depersonalization (DEP).*

### 2.8. Impact of Burnout on Job Satisfaction and Job Performance

Hotel employees directly serve customers at service encounters. Employees may therefore experience substantial psychological or physical pain when processing the various demands of customers. As such, the burnout and stress that hotel employees experience poses a serious risk and can cause negative results, such as decreased job satisfaction, absenteeism, and turnover [41]. In addition, employees who are experiencing difficult psychological situations, such as emotional exhaustion, reduced personal accomplishment, and depersonalization, which are the subordinate factors of burnout, are not proactive in communications with customers [54], and such non-proactive and cynical responses can lead to decreased service quality and negative attitudes or behaviors toward customers [54,55]. These results of prior research imply that burnout is a risk factor for job satisfaction [44]. That is, experiencing burnout decreases employee satisfaction, creates passive employee attitudes, and reduces employee commitment. Job satisfaction is a very important factor in the positive behavior of employees. According to Arnett et al. [56], research on the effect of job satisfaction on the positive behavior of employees indicates that when employees are satisfied with their jobs, overall job performance improves, including customer service improvements, improved relationships with fellow employees, and increased organizational commitment. Thus, job satisfaction has a high probability of improving job performance through the employee’s active will, passion toward the job, and commitment; furthermore, increased job satisfaction can lead to improvements in the profitability and productivity of the organization. Therefore, in this study, we have set the following hypotheses based on prior research.

**Hypothesis 7** **(H7).** *Emotional exhaustion (EE) will have a negative effect on job satisfaction (JS).*

**Hypothesis 8** **(H8).** *Reduced personal accomplishment (RPA) will have a negative effect on job satisfaction (JS).*

**Hypothesis 9** **(H9).** *Depersonalization (DEP) will have a negative effect on job satisfaction (JS).*

**Hypothesis 10** **(H10).** *Job satisfaction (JS) will have a positive effect on job performance (JP).*

### 2.9. Gender Difference

Gender is a very important variable in the studies related to the service industry [57]. Among demographic characteristics, gender can be easily confirmed in most situations, and it can provide even greater results for providing better services or establishing strategies [58]. Many studies commonly utilize the social role theory to describe gender differences [59]. According to the social role theory, because men and women are socialized in different ways and have different roles in society, they exhibit different behaviors [59]. More specifically, men are more proactive and self-directed than women, and in particular, tend to take on risk [60]. In addition, women have greater avoidant tendencies than men have [61]. Clearly, such differences in disposition may elicit different reactions to indoor and outdoor environments.

Men and women also appear to differ in their attitudes and reactions towards nature-friendly environments. Tamosiunas et al. [62] argue that women use nature-friendly environments more actively than men do and receive more benefits (e.g., mental health and stress recovery) from these environments. In contrast, Richardson and Mitchell [63] argue that men use green spaces more than women do and receive more benefits from them. Therefore, we can see men’s and women’s attitudes, behaviors, and reactions to nature-friendly environments are different. In addition, men’s and women’s attitudes and responses to burnout differ. According to the existing research, women experience greater stress than men and show stronger negative emotions [64,65]. Therefore, in this study, we set the following hypothesis based on the theoretical inferences discussed.

**Hypothesis** **11a.** *Gender will have a moderating role in the relationship between specially designed green places and spaces (SDGPs) within hotels and emotional exhaustion (EE).*

**Hypothesis** **11b.** *Gender will have a moderating role in the relationship between specially designed green places and spaces (SDGPs) within hotels and reduced personal accomplishment (RPA).*

**Hypothesis** **11c.** *Gender will have a moderating role in the relationship between specially designed green places and spaces (SDGPs) within hotels and depersonalization (DEP).*

**Hypothesis** **11d.** *Gender will have a moderating role in the relationship between existing natural environments (ENE) and emotional exhaustion (EE).*

**Hypothesis** **11e.** *Gender will have a moderating role in the relationship between existing natural environments (ENE) and reduced personal accomplishment (RPA).*

**Hypothesis** **11f.** *Gender will have a moderating role in the relationship between existing natural environments (ENE) and depersonalization (DEP).*

**Hypothesis** **11g.** *Gender will have a moderating role in the relationship between emotional exhaustion (EE) and job satisfaction (JS).*

**Hypothesis** **11h.** *Gender will have a moderating role in the relationship between reduced personal accomplishment (RPA) and job satisfaction (JS).*

**Hypothesis** **11i.** *Gender will have a moderating role in the relationship between depersonalization (DEP) and job satisfaction (JS).*

**Hypothesis** **11j.** *Gender will have a moderating role in the relationship between job satisfaction (JS) and job performance (JP).*

### 2.10. Proposed Model

In this study, we set eleven hypotheses appropriate to the purpose of this study based on prior research. We verified the moderating effect of gender in the variables presented in this research by testing hypothesis 11a–j.

## 3. Methods

### 3.1. Measurement and Questionnaire Development

The questions in this study’s questionnaire are largely divided into three categories (i.e., a description of the research, questions related to variables, and information on demographic characteristics). The 28 measurement items used in this study were based on questions with proven validity in existing research [50,66,67] that were modified and supplemented to fit this research (see Appendix A). Specifically, the hotel’s green environment was defined as the green space specially designed in the hotel and the natural environment (e.g., mountains, rivers, seas, lakes) around the hotel; and eight questions based on the studies of Bosch and Sang [10], Pietilä et al., [5], and Vujcic et al. [18] were used. In addition, burnout was defined as emotional depletion, lack of achievement, and depersonalization; 12 questions based on the study by Karatepe and Uludag [50] were used. Lastly, job satisfaction was defined as the positive emotional state of the job perceived by the employee, job performance was defined as the achievement of the goals and the differentiation from competitors as perceived by the employees and the company, and each was measured using four questions. We used a seven-point Likert-type scale ranging from very not so (1) to very much so (7), and we used the commonly utilized multi-item structure to measure variables. More specifically, four questions were used for each of the variables presented in this study. In addition, the initial survey for this research was conducted through a pretest on three groups each composed of eight people (i.e., university professors whose research focuses on the human resource management of hotel corporations, management level employees working in five-star hotels, and graduate students majoring in hotel management). After conducting pretests on these three groups, we modified and supplemented the questionnaire so that it could be accurately understood by hotel employees.

### 3.2. Data Collection and Sample Profile

To collect the data used in this study’s empirical analysis, we utilized a system from an internet research company to conduct a web-based questionnaire. A brief explanation on the purpose of this questionnaire was distributed to section managers in eleven five-star hotels located in Seoul, the capital of South Korea, and the capital region. Before distributing the questionnaire, we explained the purpose of the study to section managers and asked them to distribute the questionnaire only to the employees in each department (e.g., the guestroom, catering, food and drink, promotion, public relations, finance, and human resources departments) who agreed to participate in this survey. In addition, the questionnaire was designed so that participants could click on the provided URL to read and reply to the questionnaire in detail.

A total of 320 samples were collected through the survey, with a response rate of 87%, and 309 valid samples were used for the empirical analysis. Through the data collection process, we were able to collect 309 observations that were judged valid for empirical analysis. The 309 respondents used in the empirical analysis included 160 men (51.8%) and 149 women (48.2%). The respondents were mainly in their 20s and 30s, with 227 people in this age group (73.4%) and 82 people (26.6%) in their 40s and 50s. The sample included 305 (98.7%) respondents with a university education or higher. In terms of employment type, 276 (89.3%) respondents were permanent employees, and 33 (10.7%) were contracted employees. We found that 116 (37.5%) respondents were staff, 87 (23.8%) were chiefs, 58 (18.8%) were assistant managers, and 48 (15.5%) were section chiefs or above. Lastly, 24 (7.8%) respondents worked in catering, 100 (32.4%) worked in Food and Beverage, 93 (30.1%) worked in guestrooms, and 47 (15.2%) worked in promotion, implying that most respondents worked in operations.

## 4. Results

### 4.1. Confirmatory Factor Analysis

Before performing an empirical analysis, the skewness and kurtosis values were examined to confirm the normality of the data. The absolute values of both the skewness and kurtosis values were less than 2, indicating that there was no problem with the normality of the data. This study’s empirical analysis was conducted using Statistical Package for the Social Sciences 22.0 and Analysis of Moment Structures 22.0 based on the collected data. To evaluate the unidimensionality of the measures and the measurement model, we conducted confirmatory factor analysis, the most widely used analysis method to evaluate the reliability and validity of measures, using the maximum likelihood estimation method. The results of the analysis are as follows. The suitability of the measurement model was within statistically acceptable levels (X^2^ = 623.474, df = 329, *p* < 0.01, X^2^/df = 1.895, RMR = 0.090, RMSEA = 0.054, CFI = 0.980, NFI = 0.959). If the average variance extracted (AVE), which verifies convergent validity, is over 0.5 and the composite reliability (CR), which verifies internal consistency, is over 0.7, we can say that the model has convergent validity [68]. The results of the empirical analysis in this study indicated AVE values between 0.704 and 0.842 and CR values between 0.877 and 0.963. Thus, the convergent validity and internal consistency of the measured variables used in the empirical analysis were verified. In addition, all the correlation coefficients shown in Table 1 have values below 0.8, and the AVE value was greater than the square of the correlation coefficient between the potential variables, implying no discriminant validity issues [68].

### 4.2. Structural Model Results and Hypotheses Testing

To evaluate the hypotheses presented in this research, we estimated structural equations. The R^2^ values of the variables presented in this research model were 0.098 (EE), 0.309 (RPA), 0.231 (DEP), 0.253 (JS), and 0.106 (JP), showing that the variables were appropriately informative. Detailed results are shown in Table 2.

The verification results of hypotheses 1–11 presented in this study are as follows. SDGP (H1: β = −0.268, *p* < 0.01/H2: β = −0.160, *p* < 0.05/H3: β = −0.187, *p* < 0.01) was shown to have a meaningful effect on EE, RPA, and DEP. ENE (H4: β = −0.068, *p* > 0.05/H5: β = −0.445, *p* < 0.01/H6: β = −0.344, *p* < 0.01) was shown to have a meaningful effect on RPA and DEP, but it did not have a meaningful effect on EE. Therefore hypotheses 1, 2, 3, 5, and 6 were supported, whereas hypothesis 4 was rejected. Then, the influencing relationship of burnout on job satisfaction was verified. The results showed that exhaustion (H7: β = −0.421, *p* < 0.01) and depersonalization (H9: β = −0.178, *p* < 0.01) had a meaningful effect on job satisfaction, whereas reduced personal accomplishment (H8: β = −0.079, *p* > 0.05) was not shown to have a meaningful effect on job satisfaction. Therefore, hypotheses 7 and 9 were supported, whereas hypothesis 8 was rejected. Finally, JS (H10: β = 0.326, *p* < 0.01) was shown to have a meaningful effect on JP, and hypothesis 10 was supported.

It is known that utilizing a mediating framework in a theoretical model can aid in the understanding of the complex relationships in the research structure [31]. Therefore, we verified the indirect effects of SDGP and ENE on JS and JP. As a result, SDGP (β _SDGP − EE/RPA/DEP − JS_ = 0.159, *p* < 0.01; β _SDGP − EE/RPA/DEP − JS − JP_ = 0.052, *p* < 0.05) and ENE (β _ENE − EE/RPA/DEP − JS_ = 0.125, *p* < 0.05; β _ENE − EE/RPA/DEP − JS − JP_ = 0.041, *p* < 0.05) were shown to have meaningful indirect effects on JS and JP. These results show that EE, RPA, and DEP have important mediating effects within the proposed conceptual framework.

### 4.3. Moderating Impact Assessment

**Figure 1:**
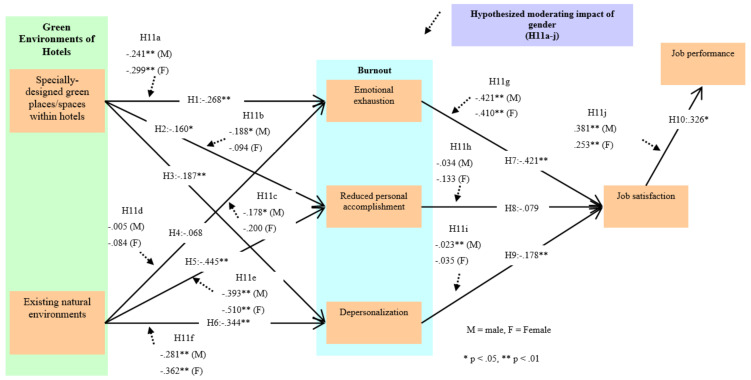
Results of the structural model estimation and baseline model assessment (*n* = 309). Goodness-of-fit statistics for the structural model: χ^2^ = 963.900, *df* = 339, *p* < 0.001, χ^2^/*df* = 2.764, RMSEA = 0.076, CFI = 0.959, IFI = 0.959, and TLI = 0.955. Goodness-of-fit statistics for the baseline model: χ^2^ = 1564.184, *df* = 699, *p* < 0.01, χ^2^/*df* = 2.238, RMSEA = 0.063, CFI = 0.942, NFI = 0.901, TLI = 0.938.

The multi-group analysis method was used to evaluate the moderating effect of gender in the research model used in this study. The results of the analysis are as follows. The differences in the chi-square values for the relationship between SDGP and burnout (EE, RPA, DEP) were not significant at the 0.05 level (Δχ^2^(1) = 0.136/0.488/0.048, *p* > 0.05). In addition, the differences in the chi-square values for the relationship between ENE and burnout (EE, RPA, DEP) were not significant at the 0.05 level (Δχ^2^(1) = 0.231/0.134/0.144, *p* > 0.05). Thus, hypotheses 11a–f were rejected. Then, we found that the differences in the chi-square values were not significant at the 0.05 level for the relationship between burnout (EE, RPA, DEP) and JS (Δχ^2^(1) = 0.001/0.415/2.888, *p* > 0.05) for any of EE, RPA, or DEP. Thus, hypotheses 11g–i were all rejected as well. Finally, the difference in the chi-square values for the relationship between JS and JP was not significant at the 0.05 level, implying that hypothesis 11j could be rejected (Δχ^2^(1) = 0.579, *p* > 0.05). Ultimately, all of the hypotheses presented to evaluate the moderating effect of gender were rejected. However, we derived a significant result through this verification of the moderating effect of gender. First, although we found that the influencing relationship of SDGP on RPA/DEP was statistically significant for men (β = −0.188, *p* < 0.05), we did not find a similar statistically significant effect for women (β = −0.094, *p* > 0.05). In addition, we found that the influencing relationship of DEP on JS was significant (β = −0.302, *p* < 0.01), but again, we did not find a statistically significant effect for women (β = −0.035, *p* > 0.05). These results demonstrate that, although the research hypotheses were rejected, the results were still very significant, as we found clear differences between women and men in each influencing relationship. The detailed results are shown in Table 3 and Figure 1.

## 5. Discussion and Implication

Our results confirmed the importance of the role of nature-friendly environments inside and outside hotels, as perceived by organization members, as well as that of job satisfaction in relation to the importance of our research structure. That is, job satisfaction and nature-friendly environments appeared to have an important effect on maximizing the job performance of hotel employees. In addition, job satisfaction appeared to have a very close relationship with the burnout perceived by hotel employees. As indicated by hypotheses 7 and 9, emotional exhaustion and depersonalization greatly decrease job satisfaction. This finding emphasizes the need to identify methods that can minimize the burnout experienced by hotel employees, prevent burnout, or allow a quick recovery from burnout. This study confirmed the fact that the nature-friendly environments perceived by hotel employees minimize burnout and aid in a quick recovery from burnout. As can be seen through hypotheses 1, 2, 3, 5, and 6, we confirmed that the specially designed green places and existing natural environments of hotels greatly decrease the burnout of hotel employees.

This study did not find a statistically significant difference between men and women in maximizing the performance of hotel employees through NBSs. That is, hypothesis 11a–j, which were analyzed based on the results of chi-square tests, were rejected. However, these analyses did find very meaningful results. First, we can conclude that the nature-friendly environments of hotels had very positive effects on all hotel employees regardless of gender. Hypothesis 11b and 11c were intended to check whether the direction of impact differed between men and women. Although the results showed that specially designed green places decreased men’s burnout by a statistically meaningful amount, burnout did not decrease for women in a statistically meaningful way. However, both men and women were confirmed to experience decreases in burnout. These results are identical to those of Vujuic et al., [18]. In their study, the effect of NBSs on mental health and well-being showed no differences between men and women in that both received positive effects, but men received a greater positive effect than women did. Therefore, we can observe that the effect of NBSs is positive regardless of gender and that men respond more positively to NBSs. Second, the analysis of the difference between men and women regarding the influencing effect of depersonalization on job satisfaction showed no difference in the direction of impact. Thus, depersonalization was confirmed to decrease job satisfaction regardless of gender, and it appeared to have a very statistically significant effect on men. This finding shows that men have a greater direct response to external environments and psychological stimuli than women do.

An examination of the existing research on environmental behavior and public health shows that although the importance of NBSs to social and environmental problems is gradually being emphasized, tests on the possible applications of NBSs for organizational behavior in the service industry have almost never been conducted. By verifying the specific role of the nature-friendly physical environment in a hotel, such as specially designed green places and existing natural environments, in the process of maximizing the job performance of hotel employees through NBSs, which has almost never been discussed in existing research, we expanded upon and successfully supplemented the gaps in the existing research. In addition, we clearly identified the complex theoretical relationship between mental disorders, such as burnout, and the nature-friendly environments of hotels, as well as the effect that these relationships have on the overall satisfaction and job performances of hotel employees. These findings, that is, the theoretical framework that includes the variables presented in this study, sufficiently describe and demonstrate the effect on the job performances of hotel employees. In addition, we reconfirmed the importance of nature-friendly environments, such as NBSs, and showed the validity of applying these concepts to the organizational behavior of hotel employees. Ultimately, this study can greatly aid hotel corporations in appreciating the importance of NBSs by demonstrating that the solutions reduce the burnout of hotel employees, improve job satisfaction, and ultimately, can improve job performance to aid in the development of the corporation. Therefore, we can say that this study provides a high level of theoretical value and implications.

When seen from a practical perspective for hotels, this study indicates the need to focus on improving the function of physical environments inside and outside hotels because the results of the analysis emphasize the importance of the nature-friendly physical environment within the hotel as well as the natural environments near the hotel. More specifically, many various nature-friendly items (e.g., flowers, potted plants, trees, phytoncide aromas, and plants) need to be placed in the environment. Doing so not only has a visual effect but also can play a positive role in the psychological comfort and mental health of hotel employees by allowing them to appreciate the scents of nature. In addition, the external natural environment of the hotel needs to be improved or upgraded. Of course, the external natural environment (e.g., mountains, rivers, lakes, air quality, forests, and parks) near hotels cannot be artificially built or developed by hotel corporations. However, the hotel can strengthen its external landscape and green space, and large windows can be installed in the building to improve the visibility of the surrounding natural environment. These proposals can be very meaningful in that they can improve the satisfaction and performance of hotel employees through NBSs given the results of this study. Therefore, improvements to the nature-friendly environments inside and outside hotels for the purpose of improving the benefits of NBSs can provide great support for the development and the psychological and physical well-being of organization members, and can also support the development and performance improvements of the hotel corporation.

The results of this study face a few limitations. First, we must be very cautious about generalizing the results to other industries, as this study was limited to employees of hotel corporations, and we must consider the unique characteristics of hotels. That is, hotels have many, varied nature-friendly physical environments because they operate in the luxury segment and in the service industry. Second, the research subjects are limited to those in South Korea, but the natural environments surrounding hotels may differ based on geographical location. Thus, we must also consider regions that have difficulty accessing natural environments. Third, focus was placed only on the responses and behavior of hotel employees. However, customer behavior in response to NBSs should also be researched to understand hotel corporation performance. Fourth, rather than using random sampling methods, only one type of information was used during the process of collecting samples. Thus, this could be improved in further research.

## 6. Conclusions

The most important findings of this research were that the nature-friendly environments of hotels decreased the burnout of hotel employees and that job satisfaction and job performance were improved due to a decrease in burnout. That is, SDGPs and ENE decreased negative factors, such as stress and depression, in hotel employees, and increased psychological and physical recovery. Furthermore, SDGPs and ENE were shown to be important methods for improving the satisfaction and performance of employees. The direct performance of hotel employees improves the performance of hotel corporations and can be a great driving force for future growth. The information presented based on the results of this study aids in understanding the positive effect of hotels’ nature-friendly environments on hotel employees and corporations. In particular, these environments minimize the burnout that occurs due to the characteristics of hotel jobs, such as the customer service process, irregular schedules, and emotional labor, and they provide great help in maximizing employee performance, which forms the foundation for the performance of a hotel corporation.

## Figures and Tables

**Table 1:** Correlation, mean, standard deviation, average variance extracted, and reliability (*n* = 309).

Variables	(a)	(b)	(c)	(d)	(e)	(f)	(g)	AVE	CR
(a) SDGP	1.000	–	–	–	–	–	–	0.704	0.905
(b) ENE	0.573 ^a^ (0.328) ^b^	1.000	–	–	–	–	–	0.642	0.877
(c) EE	−0.303 (0.091)	−0.232 (0.053)	1.000	–	–	–	–	0.777	0.949
(d) RPA	−0.413 (0.170)	−0.507 (0.257)	0.393 (0.154)	1.000	–	–	–	0.832	0.963
(e) DEP	−0.373 (0.139)	−0.433 (0.187)	0.483 (0.233)	0.756 (0.571)	1.000	–	–	0.749	0.940
(f) JS	0.399 (0.159)	0.280 (0.078)	−0.511 (0.261)	−0.365 (0.133)	−0.418 (0.174)	1.000	–	0.778	0.949
(g) JP	0.248 (0.061)	0.370 (0.136)	−0.245 (0.060)	−0.386 (0.148)	−0.366 (0.133)	0.335 (0.112)	1.000	0.842	0.966
Mean	5.381	5.373	3.698	2.208	2.623	4.609	5.796		
SD	1.542	1.306	2.045	1.590	1.778	2.607	1.414		

Note 1: SDGP = specially designed green place, ENE = existing natural environments, EE = emotional exhaustion, RPA = reduced personal accomplishment, DEP = depersonalization, JS = job satisfaction, JP = job performance. Note 2: Goodness-of-fit statistics: χ^2^ = 623.474, *df* = 329, *p* < 0.01, χ^2^/*df* = 1.895, SRMR = 0.060, RMRRMSEA = 0.054, CFI = 0.980, NFI = 0.959, TLI = 0.977, ^a^ Correlations, ^b^ Squared correlations.

**Table 2:** Coefficient, t-value, total impact, indirect impact, R^2^, and hypotheses testing (*n* = 309).

Proposed Paths				β	*t*-Values
H1	SDGP	→	EE	−0.268	−3.749 **
H2	SDGP	→	RPA	−0.160	−2.527 **
H3	SDGP	→	DEP	−0.187	−2.823 **
H4	ENE	→	EE	−0.068	−0.945
H5	ENE	→	RPA	−0.445	−6.615 **
H6	ENE	→	DEP	−0.344	−4.998 **
H7	EE	→	JS	−0.421	−8.218 **
H8	RPA	→	JS	−0.079	−1.495
H9	DEP	→	JS	−0.178	−3.390 **
H10	JS	→	JP	0.326	5.925 **
Total impact on JP β SDGP = 0.052 ** β ENE = 0.041 * β EE = −0.137 ** β RPA = −0.026 β DEP = −0.058 β JS = 0.326 **	Total variance explained (R^2^): R^2^ for EE = 0.098 R^2^ for RPA = 0.309 R^2^ for DEP = 0.231 R^2^ for JS = 0.253 R^2^ for JP = 0.106 Goodness-of-fit statistics for the structural model: χ^2^ = 963.900, *df* = 339, *p* < 0.001, χ^2^/*df* = 2.764, RMSEA = 0.076, CFI = 0.959, IFI = 0.959, and TLI = 0.955			Indirect impact: β _SDGP − EE/RPA/DEP − JS_ = 0.159 ** β _ENE − EE/RPA/DEP − JS_ = 0.125 ** β _SDGP − EE/RPA/DEP − JS_ = 0.052 * β _ENE − EE/RPA/DEP − JS_ = 0.041 * * *p* < 0.05 and ** *p* < 0.01	

* *p* < 0.05 and ** *p* < 0.01.

**Table 3:** Results of the invariance tests for structural models (*n* = 309).

Paths	Male (*n* = 160)		Male (*n* = 160)		Baseline Model	Nested Model
	β	*t*-Value	β	*t*-Value	(Freely Estimated)	(Constrained to be Equal)
H1a: SDGP → EE	−0.241	−2.716 **	−0.299	−2.533 **	χ^2^ (699) = 1564.184	χ^2^ (700) = 1564.320 ^a^
H1b: SDGP → RPA	−0.188	−2.356 *	−0.094	−0.899	χ^2^ (699) = 1564.184	χ^2^ (700) = 1564.672 ^b^
H1c: SDGP → DEP	−0.178	−2.102 *	−0.200	−1.831	χ^2^ (699) = 1564.184	χ^2^ (700) = 1564.232 ^c^
H1d: ENE → EE	−0.005	−0.061	−0.084	−0.703	χ^2^ (699) = 1564.184	χ^2^ (700) = 1564.415 ^d^
H1e: ENE → RPA	−0.393	−4.773 **	−0.510	−4.683 **	χ^2^ (699) = 1564.184	χ^2^ (700) = 1564.318 ^e^
H1f: ENE → DEP	−0.281	−3.175 **	−0.362	−3.258 **	χ^2^ (699) = 1564.184	χ^2^ (700) = 1564.328 ^f^
H1g: EE → JS	−0.421	−6.172 **	−0.410	−5.375 **	χ^2^ (699) = 1564.184	χ^2^ (700) = 1564.185 ^g^
H1h: RPA → JS	−0.034	−0.488	−0.133	−1.680	χ^2^ (699) = 1564.184	χ^2^ (700) = 1564.599 ^h^
H1i: DEP → JS	−0.302	−4.367 **	−0.035	−0.439	χ^2^ (699) = 1564.184	χ^2^ (700) = 1567.072 ^i^
H1j: JS → JP	0.381	5.095 **	0.253	3.137 **	χ^2^ (699) = 1564.184	χ^2^ (700) = 1564.763 ^j^
Chi-square test: ^a^ Δχ^2^ (1) = 0.136, *p* > 0.05 ^b^ Δχ^2^ (1) = 0.488, *p* > 0.05 ^c^ Δχ^2^ (1) = 0.048, *p* > 0.05 ^d^ Δχ^2^ (1) = 0.231, *p* > 0.05 ^e^ Δχ^2^ (1) = 0.134, *p* > 0.05 ^f^ Δχ^2^ (1) = 0.144, *p* > 0.05 ^g^ Δχ^2^ (1) = 0.001, *p* > 0.05 ^h^ Δχ^2^ (1) = 0.415, *p* > 0.05 ^i^ Δχ^2^ (1) = 2.888, *p* > 0.05 ^j^ Δχ^2^ (1) = 0.579, *p* > 0.05		Hypotheses testing: H11a: Not supported H11b: Not supported H11c: Not supported H11d: Not supported H11e: Not supported H11f: Not supported H11g: Not supported H11h: Not supported H11i: Not supported H11j: Not supported		Goodness-of-fit statistics for the baseline model: χ^2^ = 1564.184, df = 699 *p* < 0.01, χ^2^/df = 2.238, RMSEA = 0.063, CFI = 0.942, NFI = 0.901, TLI = 0.938 * *p* < 0.05, ** *p* < 0.01		

SDGP = specially designed green place, ENE = existing natural environments, EE = emotional exhaustion, RPA = reduced personal accomplishment, DEP = depersonalization, JS = job satisfaction, JP = job performance.

## Appendix A

**Green Environments of Hotels**
- I can easily see a variety of living plants and green interior decorations in the lobby area of this hotel. - Diverse green items and light through glass windows are easily observable in the restaurants (e.g., staff cafeteria) of this hotel. - A variety of flowers, trees, and potted plants are placed in the lounge of this hotel. - Green space(s) is easily accessible throughout this hotel. - This hotel is located close to natural environments (e.g., mountains, rivers, forests, lakes, natural parks). - The weather (e.g., temperature, humidity, and precipitation) of the region surrounding this hotel is very good. - The quality of air (e.g., dust, ozone concentration) of the region surrounding this hotel is very good. - The region around this hotel is safe from natural disasters (e.g., earthquakes, typhoons, tsunamis, floods).
**Burnout**
- I feel emotionally drained from my work. - I feel used up at the end of the workday. - I feel fatigued when I get up in the morning and have to face another day on the job. - I feel burned out from my work.I don’t feel very energetic. - I don’t feel very energetic. - I don’t feel exhilarated after working closely with my customers. - I have not accomplished many worthwhile things in this job. - In my work, I don’t deal with emotional problems very calmly. - I feel I treat some customers as if they were impersonal objects. - I have become more callous towards people since I took this job. - I worry that this job is hardening me emotionally. - I do not really care what happens to some customers.
**Job Satisfaction**
- I like my salary or wages. - I like my opportunities for advancement with this hotel. - I feel satisfied with my overall job. - I find real enjoyment in my work.
**Job Performance**
- I am in the top 10% of employees here. - I get along better with customers than do others. - I know more about services delivered to customers than others. - I know what my customers expect better than others.
